# Microsatellite instability and survival after adjuvant chemotherapy among stage II and III colon cancer patients: results from a population‐based study

**DOI:** 10.1002/1878-0261.12611

**Published:** 2020-01-07

**Authors:** Elizabeth Alwers, Lina Jansen, Hendrik Bläker, Matthias Kloor, Katrin E. Tagscherer, Wilfried Roth, Daniel Boakye, Esther Herpel, Carsten Grüllich, Jenny Chang‐Claude, Hermann Brenner, Michael Hoffmeister

**Affiliations:** ^1^ Division of Clinical Epidemiology and Aging Research German Cancer Research Center (DKFZ) Heidelberg Germany; ^2^ Medical Faculty University of Heidelberg Germany; ^3^ Department of General Pathology Institute of Pathology Charité University Medicine Hospital Berlin Germany; ^4^ Department of Applied Tumor Biology Institute of Pathology University of Heidelberg Germany; ^5^ Institute of Pathology University Hospital Heidelberg Germany; ^6^ Institute of Pathology University Medical Center Mainz Germany; ^7^ NCT Tissue Bank National Center for Tumor Diseases (NCT) Heidelberg Germany; ^8^ Department of Medical Oncology National Center for Tumor Diseases University Hospital Heidelberg Germany; ^9^ Division of Cancer Epidemiology German Cancer Research Center (DKFZ) Heidelberg Germany; ^10^ Genetic Tumor Epidemiology Group University Medical Center Hamburg‐Eppendorf Germany; ^11^ Division of Preventive Oncology German Cancer Research Center (DKFZ) National Center for Tumor Diseases (NCT) Heidelberg Germany; ^12^ German Cancer Consortium (DKTK) German Cancer Research Center (DKFZ) Heidelberg Germany

**Keywords:** adjuvant chemotherapy, colon cancer, microsatellite instability, survival

## Abstract

Previous studies have reported conflicting results regarding the benefit of administering 5‐FU‐based chemotherapy to colon cancer (CC) patients with microsatellite‐instable (MSI‐high) tumors, and results from stage‐specific analyses are scarce. Patients with stage II or III CC were recruited as part of a population‐based study between 2003 and 2015. The Cox regression models including propensity score weighting were used to calculate hazard ratios and confidence intervals for the association between chemotherapy and cancer‐specific (CSS), relapse‐free (RFS), and overall survival (OS) by stage of disease and MSI status of the tumor. Median follow‐up was 6.2 years. A total of 1010 CC patients were included in the analysis (54% stage II, 46% stage III, 20% MSI‐high). Adjuvant chemotherapy was administered to 48 (8.7%) stage II and 366 (79%) stage III patients. Overall, patients who received adjuvant chemotherapy had better CSS [HR = 0.65 (0.49–0.86)] than those who received surgery alone. Among stage II patients, only 64 (12%) cancer‐related deaths occurred, none of which in MSI‐high patients who received chemotherapy. Patients with MSI‐high tumors who received adjuvant treatment showed better CSS and a tendency toward better RFS compared to MSI‐high patients who did not receive chemotherapy [HR_CSS_ = 0.36 (0.15–0.82), HR_RFS_ = 0.49 (0.22–1.06)]. Patients with microsatellite‐stable (MSS) tumors receiving adjuvant chemotherapy also had significantly better survival [HR_CSS_ = 0.65 (0.48–0.87) and HR_RFS_ = 0.68 (0.52–0.88)]. In this population‐based study including stage II and III CC patients, we observed a survival benefit of adjuvant chemotherapy for both MSS and MSI‐high tumors. Adjuvant chemotherapy seemed to be beneficial among high‐risk stage II patients with MSI‐high tumors.

## Graphical abstract

In this population‐based study including colon cancer patients with stage II and III disease, a survival benefit of adjuvant chemotherapy was observed for both microsatellite stability and microsatellite‐instable (MSI)‐high tumors. Adjuvant chemotherapy seemed to also be beneficial for high‐risk stage II patients with MSI‐high tumors.

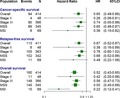

**Abbreviations**
CC: colon cancer
CI: confidence interval
CSS: cancer‐specific survival
DFS: disease‐free survival
HR: hazard ratio
MSI: microsatellite instability
MSS: microsatellite stability
OS: overall survival
PS: propensity score
RFS: relapse‐free survival

## 1 Introduction

Colorectal cancer is a leading cause of cancer incidence and mortality worldwide (Bray *et al.*, 2018). For stage III colon cancer (CC) patients, the standard of treatment includes curative resection and adjuvant chemotherapy (National Comprehensive Cancer Network, 2019). For stage II CC patients, administration of adjuvant chemotherapy is recommended only when high‐risk features are present: T4 tumors; inadequate lymph node sampling (< 12); poorly differentiated histology [for microsatellite‐instable (MSI)‐H]; lymphovascular or perineural invasion; bowel obstruction; localized perforation; or close, indeterminate, or positive margins (National Comprehensive Cancer Network, 2019).

Current guidelines recommend that stage II patients with MSI‐high tumors should not receive adjuvant treatment with 5‐FU chemotherapy (National Comprehensive Cancer Network, 2019). This recommendation was based on evidence from two clinical trials that found no survival benefit in MSI‐high tumors receiving adjuvant 5‐FU (Ribic *et al.*, 2003; Sargent *et al.*, 2010). However, other studies found no evidence for MSI status to be predictive of either beneficial or harmful effects of chemotherapy (Bertagnolli *et al.*, 2011; Hutchins *et al.*, 2011), and evidence from two trials (Klingbiel *et al.*, 2015; Quasar Collaborative Group, 2007) and a large population‐based analysis (Casadaban *et al.*, 2016) suggested that adjuvant treatment in this group of patients was beneficial. In stage III patients, the role of the MSI‐high status as a predictive biomarker is also not completely clear (Klingbiel *et al.*, 2015; Sinicrope *et al.*, 2013). A recent pooled analysis of data from the N0147 and PETACC‐8 trials showed a small but significant decrease in the risk of recurrence for MSI‐high tumors among stage III CC patients receiving adjuvant chemotherapy (Zaanan *et al.*, 2018).

Various studies have attempted to clarify these associations, but controversies still exist about the usefulness of different molecular markers in predicting survival in the context of adjuvant chemotherapy for CC. In this study, we describe the response to adjuvant chemotherapy in stage II and III CC patients and provide analyses according to the MSI status of the tumor.

## 2 Materials and methods

### 2.1 Study population

The DACHS population‐based study was conducted in 22 hospitals of the Rhine–Neckar region in Germany. Details of the study have been previously described (Blaker *et al.*, 2019). Between 2003 and 2015, patients older than 30 years of age, with a histologically confirmed diagnosis of CRC, and able to participate in an interview were recruited. Long‐term follow‐up was performed at 3, 5, and 10 years after diagnosis, including information on type of therapy, comorbidities, and recurrence of disease. Vital status, and date and cause of death were determined from population registries and death certificates issued by the health authorities. Only stage II and III CC patients were included in this analysis. Patients with rectal cancer, and stage I or IV tumors, missing information on stage or MSI status, who died in the first month after surgery, and who received neoadjuvant chemotherapy were excluded from this analysis. Figure S1 presents the selection process and corresponding number of excluded patients. The study was approved by the ethics committees of the University of Heidelberg and of the Medical Chambers of Baden‐Württemberg and Rhineland‐Palatinate, and all participants signed an informed consent.

### 2.2 Marker characterization

Tumor tissue analyses were performed on formalin‐fixed, paraffin‐embedded samples. MSI status was determined using a mononucleotide marker panel (BAT25, BAT26, CAT25; Findeisen *et al.*, 2005) or as reported from the patient’s medical records for 93% and 7% of patients, respectively. Determination of MSI using the triple‐marker panel has been shown to correctly classify 100% of MSI‐high cases when compared to the traditional five‐marker panel recommended by the National Cancer Institute (BAT25, BAT26, D2S123, D5S346, and D17S250; Findeisen *et al.*, 2005). The MSI status extracted from pathology reports (7% of patients) was mostly determined using immunohistochemistry for MLH1, MSH2, or MSH6, or by genetic testing.

### 2.3 Adjuvant chemotherapy

All patients underwent surgical resection of the tumor. The information on adjuvant chemotherapy and administered scheme was reported by the treating physician during follow‐up. The type of chemotherapy administered was categorized as ‘FU‐based’ when the reported treatment included either 5‐FU or capecitabine alone or 5‐FU+ leucovorin, and as ‘oxaliplatin‐based’ when it included oxaliplatin in addition to 5‐FU/leucovorin (FOLFOX), or in addition to capecitabine (CAPOX, XELOX). Due to low sample sizes in some of the specific chemotherapy schemes, the main exposure variable used in the analyses remained as having received adjuvant chemotherapy vs surgery alone.

### 2.4 Statistical analyses

Clinical and pathological characteristics were described for the entire study population and by the treatment group (adjuvant chemotherapy vs no chemotherapy). The Kaplan–Meier plots were computed to estimate survival curves stratified by stage and MSI status and to calculate 5‐year survival rates and log‐rank tests. Cancer‐specific survival (CSS) was defined as time from diagnosis until death from CC, relapse‐free survival (RFS) from diagnosis until reappearance of disease, metastases, cancer death or death from other causes, and overall survival (OS) until death from any cause.

To account for the observational nature of the study, propensity scores (PSs) were calculated and used to correct for the covariate imbalance between the treated and untreated groups (Brookhart *et al.*, 2013; Sturmer *et al.*, 2014). PSs were estimated using logistic regression by modeling the treatment status as a function of potential confounders including age, sex, proximal or distal location, MSI status, histological grade, stage, number of affected and examined lymph nodes, and presence of comorbidities. The Charlson comorbidity index was used to calculate an overall comorbidity score to group patients into four categories from score 0 (no comorbidities) to score 3 (severe comorbidities) (Charlson *et al.*, 1987; Quan *et al.*, 2005).

Propensity scores were then used to weight the study population to estimate the average treatment effect on the treated (ATT: 1 for the treated group and PS/[1‐PS] for the control group; Austin, 2014). Balance of potential confounders between treated and untreated groups was assessed (Austin and Stuart, 2015): (a) by graphical inspection of the overlapping distributions of PS before and after adjustment; and (b) by calculating absolute mean differences for each covariate, where a threshold of 0.10 was considered to indicate residual imbalance between exposure groups (Nguyen *et al.*, 2017). PS models were refitted independently in subgroup analyses for stage II, stage III, microsatellite stability (MSS), and MSI‐high patients to improve residual balance, to exclude the variable used for stratification when calculating the PS (i.e., stage or MSI status), and to account for the different characteristics considered by clinicians in the decision of whether to administer chemotherapy to stage II and III patients. PS‐weighted Cox proportional hazards models were used to estimate hazard ratios (HRs) and confidence intervals (95% CIs) for the overall study population and for each subgroup. Due to the small sample size in subgroups of combined stage and MSI status (i.e., stage II MSS, stage II MSI), balance after PS weighting was not achieved. Therefore, only descriptive statistics are presented for these subgroups. Also, because of the limited number of patients in the MSI subgroup, no test for interaction of MSI status and chemotherapy was performed with respect to survival. All statistical analyses were performed using sas version 9.4 (SAS Institute Inc., Cary, NC, USA) and r version 3.5.1 software (R Core Team, 2018).

### 2.5 Meta‐analysis of results

Given the low number of patients with stage II MSI‐high cancers who received chemotherapy, a meta‐analysis was performed to compare the results obtained from the survival analysis in the subgroup of stage II MSI‐high patients with previously reported literature. Results from studies reporting survival for stage II colon or colorectal cancer patients stratified by MSI status were included. These studies were identified using a combination of search terms (see Data S1) in PubMed and by manual search of the bibliographies from relevant studies. The search strategy aimed to identify studies reporting HR and 95% CI for survival outcomes [i.e., disease‐free survival (DFS) or OS] for adjuvant chemotherapy compared to no chemotherapy (surgery alone). Clinical trials comparing the addition of a chemotherapy agent to an already existing treatment were not included in this meta‐analysis.

## 3 Results

### 3.1 Overall characteristics

One thousand and ten CC patients were included in this analysis, of which 54% (*n* = 549) and 46% (*n* = 461) had stage II and III disease, respectively. Overall, 41% (*n* = 414) received adjuvant chemotherapy, 88% (*n* = 366) of whom were stage III. Table 1 presents the baseline characteristics of the study population overall and by adjuvant chemotherapy status. Patients who received chemotherapy were younger and had a lower comorbidity index compared to those not receiving adjuvant treatment. Overall, 20% (*n* = 206) of patients had MSI‐high tumors, a third (*n* = 69) of whom received adjuvant chemotherapy.

**Table 1:** Characteristics of stage II and III CC patients by adjuvant chemotherapy. Proximal colon: cecum to splenic flexure; distal colon: from splenic flexure to sigmoid. *P*‐values from chi‐square tests.

Variable	*n* = 1010 (%)	Received adjuvant chemotherapy		
		No	Yes	*P*‐value
		*n* = 596	*n* = 414	
Age				
< 65	305 (30.2)	145 (24.3)	160 (38.6)	< 0.001
65–74	341 (33.8)	186 (31.2)	155 (37.4)	
> 75	364 (36.0)	265 (44.5)	99 (23.9)	
Gender				
Female	479 (47.4)	280 (47.0)	199 (48.1)	0.782
Male	531 (52.6)	316 (53.0)	215 (51.9)	
Location				
Proximal	585 (57.9)	356 (59.7)	229 (55.3)	0.182
Distal	425 (42.1)	240 (40.3)	185 (44.7)	
Family history				
No	858 (85.0)	508 (85.2)	350 (84.5)	0.831
Yes	152 (15.0)	88 (14.8)	64 (15.5)	
Comorbidity index				
0	567 (56.1)	301 (50.5)	266 (64.3)	< 0.001
1	197 (19.5)	128 (21.5)	69 (16.7)	
2	139 (13.8)	94 (15.8)	45 (10.9)	
3	107 (10.6)	73 (12.2)	34 (8.2)	
Stage at diagnosis				
II	549 (54.4)	501 (84.1)	48 (11.6)	< 0.001
III	461 (45.6)	95 (15.9)	366 (88.4)	
T stage				
1–2	49 (4.9)	8 (1.3)	41 (9.9)	< 0.001
3	830 (82.2)	540 (90.6)	290 (70.0)	
4	131 (13.0)	48 (8.1)	83 (20.0)	
N stage				
0	549 (54.4)	501 (84.1)	48 (11.6)	< 0.001
1	293 (29.0)	65 (10.9)	228 (55.1)	
2	168 (16.6)	30 (5.0)	138 (33.3)	
MSI status				
MSS	804 (79.6)	459 (77.0)	345 (83.3)	0.018
MSI‐high	206 (20.4)	137 (23.0)	69 (16.7)	
Grade				
1–2	697 (69.0)	439 (73.7)	258 (62.3)	0.0002
3–4	313 (31.0)	157 (26.3)	156 (37.7)	

FU‐based or oxaliplatin‐based treatment was administered to 200 (31 stage II, 169 stage III) and 203 (14 stage II, 189 stage III) patients, respectively. The distribution of patients by chemotherapy scheme is presented in Table 2.

**Table 2:** Chemotherapy regimen used by stage of disease and MSI status—*n* (%). FU‐based, 5‐FU or capecitabine alone or in combination with leucovorin; oxaliplatin‐based: FOLFOX, XELOX, CAPOX.

	Stage II		Stage III	
	MSS	MSI‐high	MSS	MSI‐high
No chemotherapy	381 (91.6)	120 (90.2)	78 (20.1)	17 (23.3)
Adjuvant chemotherapy				
FU‐based	24 (5.7)	7 (5.3)	143 (36.9)	26 (35.6)
Oxaliplatin‐based	9 (2.2)	5 (3.8)	159 (41.0)	30 (41.1)
Other scheme	1 (0.2)	1 (0.8)	5 (1.3)	0
Scheme unknown	1 (0.2)	0	3 (0.8)	0

### 3.2 Propensity score balance

The balance plots and calculated mean differences for the adjusted populations showed improved balance between the exposure groups for all variables, both in the overall population and in the subgroup analyses (Fig. S2).

### 3.3 Cancer‐specific and relapse‐free survival

Median follow‐up time was 6.2 years. Overall, 42% (*n* = 429) of patients died, including 183 (43%) who died of CC. Cancer‐specific mortality occurred in 12% (*n* = 64) and 26% (*n* = 119) of stage II and III patients, respectively. Relapse events occurred in 16% (*n* = 89) and 30% (*n* = 140) of stage II and III patients, respectively.

Results from the Cox regression models using propensity score weighting showed significantly better CSS and RFS for patients who received chemotherapy [HR = 0.65 (0.49–0.86) and HR = 0.67 (0.52–0.87), respectively] compared to those who were treated with surgery alone (Table 3). Among stage II patients receiving adjuvant treatment, only four cancer deaths (8%) and eight relapse events (17%) occurred; significantly better CSS and RFS were observed for this subgroup. Also among stage III patients, better CSS [HR = 0.74 (0.55–0.99)] and RFS [HR = 0.78 (0.60–1.02)] were observed for those receiving adjuvant treatment compared to surgery alone. Patients with MSS tumors who received adjuvant chemotherapy had significantly better CSS [HR = 0.65 (0.48–0.87)] and RFS [HR = 0.68 (0.52–0.88)] than those treated with surgery alone. Patients with MSI‐high tumors also benefited from adjuvant chemotherapy in terms of CSS [HR = 0.36 (0.15–0.82)] and RFS [HR = 0.49 (0.22–1.06)]; the number of treated patients (*n* = 69) and events (eight cancer deaths, 11 relapse events) were low in this subgroup.

**Table 3:** Propensity score‐weighted Cox regression analysis among stage II and III CC patients overall, and by stage and MSI status. *P*‐values from likelihood‐ratio tests.

	CSS					RFS^a^					OS				
	*N*	Events	HR	95% CI	*P*‐value	*N*	Events	HR	95% CI	*P*‐value	*N*	Events	HR	95% CI	*P*‐value
Overall (*n* = 1010)															
No chemotherapy	596	89	1			591	116	1			596	269	1		
Adjuvant chemotherapy	414	94	0.65	0.49–0.86	0.002	411	113	0.67	0.52–0.87	0.002	414	160	0.52	0.43–0.65	< 0.0001
Stage II patients (*n* = 549)															
No chemotherapy	501	60	1			497	81	1			501	196	1		
Adjuvant chemotherapy	48	4	0.26	0.08–0.81	0.020	48	8	0.38	0.16–0.89	0.020	48	11	0.42	0.20–0.88	0.020
Stage III patients (*n* = 461)															
No chemotherapy	95	29	1			94	35	1			95	73	1		
Adjuvant chemotherapy	366	90	0.74	0.55–0.99	0.040	363	105	0.78	0.60–1.02	0.070	366	149	0.58	0.47–0.73	< 0.0001
MSS tumors (*n* = 804)															
No chemotherapy	459	78	1			455	101	1			459	212	1		
Adjuvant chemotherapy	345	86	0.65	0.48–0.87	0.003	342	102	0.68	0.52–0.88	0.003	345	145	0.55	0.45–0.69	< 0.0001
MSI‐high tumors (*n* = 206)															
No chemotherapy	137	11	1			136	15	1			137	57	1		
Adjuvant chemotherapy	69	8	0.36	0.15–0.82	0.010	69	11	0.49	0.22–1.06	0.070	69	15	0.22	0.12–0.41	< 0.0001

a RFS estimates based on 1002 patients.

### 3.4 Overall survival

Death from any cause occurred in 38% (*n* = 207) and 48% (*n* = 222) of stage II and III patients, respectively. In propensity score‐weighted Cox regression models, patients who received adjuvant chemotherapy had significantly better OS compared to those treated with surgery alone. In subgroup analyses, the survival benefit was maintained for both stage II and III patients and patients with MSS and MSI tumors (Table 3). MSI patients who received chemotherapy were younger than those who did not receive adjuvant treatment, and despite the inclusion of age in the PS model, this may explain the OS benefit.

### 3.5 Relapse‐free survival by combinations of stage and MSI status

The number of treated patients and deaths or relapse events by subgroups combining stage and MSI status is presented in Table 4. Among stage II patients with MSI‐high tumors who received adjuvant treatment (*n* = 13), no cancer deaths and two relapse events were observed. Multivariable Cox models in this subgroup of patients were used only to compare results with other studies in a meta‐analysis; however, the low number of events precludes these results from generating any conclusions. Among stage II MSS patients who received adjuvant treatment, also few deaths and relapse events were observed. Similar to what was observed in the stage‐specific analyses, stage III patients with MSS tumors who received adjuvant chemotherapy had better survival compared to those treated with surgery alone. Among stage III patients with MSI‐high tumors (*n* = 73), the proportion of deaths and relapse events was lower for those who received adjuvant chemotherapy.

**Table 4:** Number of events by chemotherapy exposure by combinations of stage and MSI status.

	*N*	Cancer‐specific deaths		Relapse events		Death from any cause	
		*n* (%)	5‐year survival^d^ (%)	*n* (%)	5‐year survival^d^ (%)	*n* (%)	5‐year survival^d^ (%)
Stage II MSS (*n* = 416)							
No chemotherapy	381	53 (13.9)	89.1	69^a^ (18.3)	83.6	153 (40.2)	77.6
Adjuvant chemotherapy	35	4 (11.4)	91.3	6 (17.1)	82.7	10 (28.6)	85.5
Stage II MSI‐high (*n* = 133)							
No chemotherapy	120	7 (5.8)	94.6	12 (10.0)	90.2	43 (35.8)	79.6
Adjuvant chemotherapy	13	0	100	2 (15.4)	92.3	1 (7.7)	100
Stage III MSS (*n* = 388)							
No chemotherapy	78	25 (32.1)	68.0	32 (41.0)	55.6	59 (75.6)	42.9
Adjuvant chemotherapy	310	82 (26.5)	80.7	96^b^ (31.3)	70.0	135 (43.5)	74.4
Stage III MSI‐high (*n* = 73)							
No chemotherapy	17	4 (23.5)	76.5	3^c^ (18.8)	81.3	14 (82.4)	37.8
Adjuvant chemotherapy	56	8 (14.3)	85.3	9 (16.1)	85.5	14 (25.0)	80.4

a From 377 patients.

b From 307 patients.

c From 16 patients.

d 5‐year survival calculated with the Kaplan–Meier estimates.

### 3.6 Meta‐analysis of results for stage II MSI‐high patients

**Figure 1:**
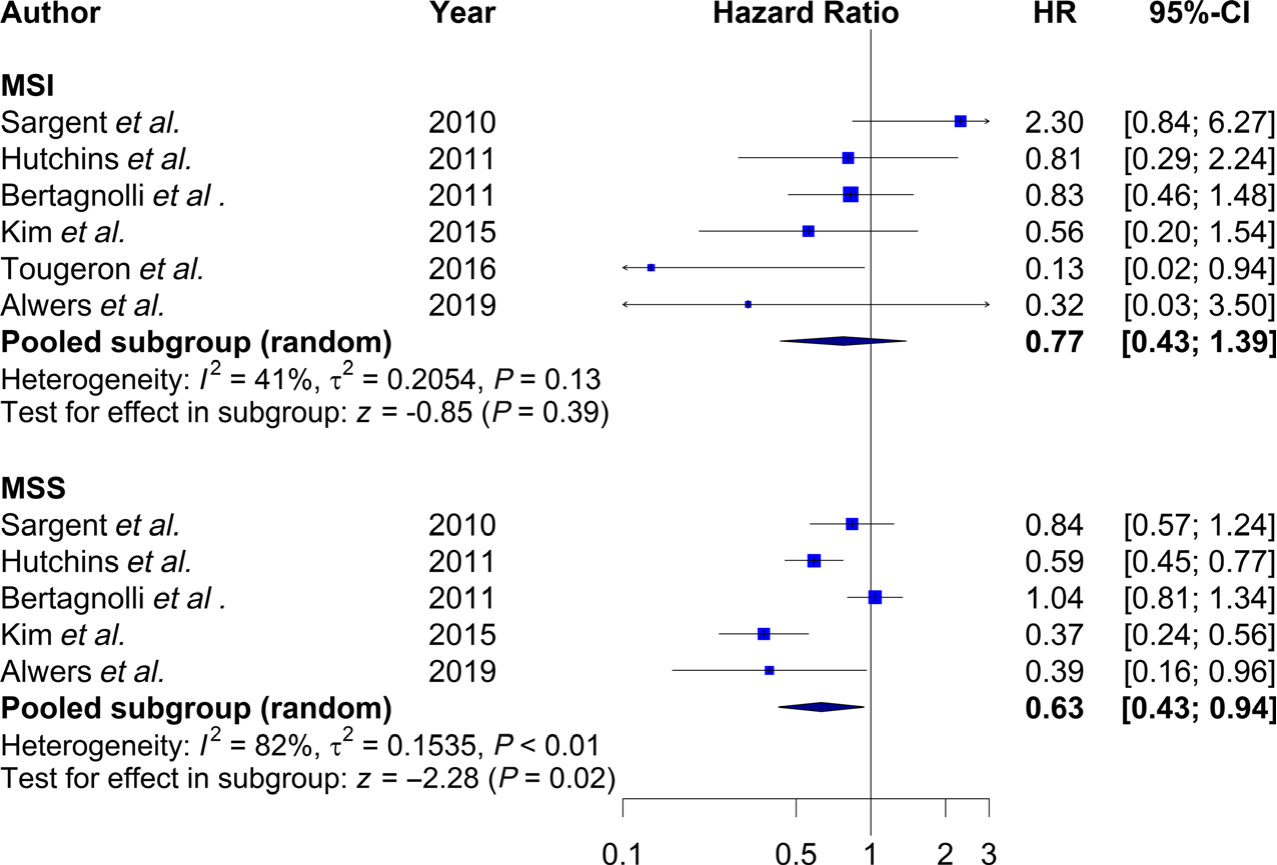
Meta‐analysis of results for DFS among stage II patients by MSI status, comparing surgery plus adjuvant chemotherapy vs surgery alone.

Characteristics of the studies included in the meta‐analysis are presented in Table S1 (Bertagnolli *et al.*, 2011; Hutchins *et al.*, 2011; Kim *et al.*, 2015; Sargent *et al.*, 2010; Tougeron *et al.*, 2016). The meta‐analysis of results including five studies for MSI‐high and four for MSS showed significantly better survival for stage II MSS patients [HR = 0.63 (0.43–0.94)] and a tendency toward better survival for stage II MSI‐high patients [HR = 0.77 (0.43–1.39)] (Fig. 1).

## 4 Discussion

In this patient cohort, we investigated the benefit of adjuvant chemotherapy in patients with CC according to disease stage and MSI status of the tumor. Overall, survival was better among patients who received adjuvant treatment compared to those who were treated with surgery alone. This reflects the known beneficial effects of adjuvant chemotherapy that have been reported in several clinical trials (Andre *et al.*, 2009; Yothers *et al.*, 2011). In subgroup analyses of stage II and III patients and patients with MSS or MSI‐high tumors, better CSS and RFS were observed.

Recent results from large clinical trials suggest that MSI‐high patients who received either 5‐FU alone or combination therapy with FOLFOX maintained their survival advantage compared to MSS patients (Klingbiel *et al.*, 2015; Zaanan *et al.*, 2018), confirming the generally better survival among MSI‐high patients and suggesting that they retain this benefit when treated with oxaliplatin‐based regimens (Andre *et al.*, 2015).

A recent systematic review (Webber *et al.*, 2015) found no difference in the survival benefit following adjuvant 5‐FU chemotherapy according to the MSI status, after including treated and untreated colorectal cancer patients. Including estimates from six studies, the pooled HR for DFS in MSS and MSI‐high cancers was 0.62 (0.54–0.71) and 0.84 (0.53–1.32), respectively; the difference between these two estimates was not statistically significant (*P* = 0.11; Webber *et al.*, 2015). However, this review did not differentiate between stage II and III and colon or rectal cancer. Our results for MSS patients are in line with those reported by the systematic review [HR = 0.68 (0.5–0.9)]; however, our results for RFS in MSI‐high patients suggest better survival [HR = 0.49 (0.2–1.1)]. This difference may be explained by the inclusion of studies on rectal cancer, whereas our study is limited to CC patients.

Few studies have reported survival results specifically for stage II MSI‐high patients, given the small number of patients who have received treatment and the resulting lack of power to detect a benefit of adjuvant treatment. Our results reflect the good prognosis that both early‐stage and MSI‐high patients have. The analysis in this small subgroup in our cohort and the comparison with other reported results suggest better survival for stage II MSI‐high patients who received adjuvant treatment compared to those treated with surgery alone. Thus, the presence of MSI‐high tumor might not be decisive for the use of chemotherapy in stage II patients with high‐risk features, especially if the option of added oxaliplatin is also considered.

Patients included in this study were recruited after 2003, when the recommendation to not administer FU‐based chemotherapy to MSI‐high patients was introduced in the international literature (Benson *et al.*, 2004; Ribic *et al.*, 2003). In Germany, this recommendation was not officially introduced until 2017; however, it is possible that clinicians in the study region modified their treatment decisions in regard to the American recommendations. Since then, the number of stage II patients with MSI‐high tumors who received chemotherapy has decreased, resulting in a low number of cases available for analyses. Nevertheless, the few deaths occurring among those patients who did receive adjuvant treatment point to a potential survival benefit. With the increasing use of combined chemotherapy, adding oxaliplatin to 5‐FU‐based regimens (i.e., FOLFOX) seems to also have a benefit in the survival of patients with MSI‐high tumors (Zaanan *et al.*, 2018). Future clinical trials will help elucidate the predictive utility of MSI and other molecular markers in the adjuvant setting for stage II and III CC patients.

The main limitations of this analysis include its observational nature and the small sample size in the stage II subgroup, since the study was not powered to detect effects of chemotherapy in small subgroups of patients. Because of the observational design of the study, the patient populations who received chemotherapy or surgery alone were not comparable at baseline. We corrected for this imbalance using propensity score weighting, which led to well‐balanced covariate distributions between patients who received chemotherapy and those treated with surgery alone, both in the overall and in stage and MSI status subgroup analyses. The possibility of residual confounding due to unmeasured factors remains and may affect the PS model assumptions. The observed results for OS were generally stronger than those observed for cancer‐specific and RFS. This reflects a real clinical practice scenario, where healthier patients are more likely to receive treatment and could be an indication that—although advanced statistical methods were employed to correct for the potential study design issues—such differences between treated and untreated patients cannot be completely removed. Information on adverse events was not taken into account in these analyses. Because the addition of oxaliplatin carries risk of adverse events, such as chronic neurological deficits (Meyers *et al.*, 2017), the decision of administering adjuvant chemotherapy to patients with MSI‐high cancers needs to be carefully considered by the clinician. Strengths of the analysis include the balance of baseline characteristics of the population to emulate a clinical trial design and the long‐term follow‐up. Additionally, our results reflect the effects of adjuvant chemotherapy in a real‐world scenario and capture changes in treatment patterns as a result of the recommendations to not administer 5‐FU to MSI patients and with the addition of oxaliplatin to the treatment schemes.

## 5 Conclusions

In conclusion, we found that adjuvant chemotherapy is beneficial for stage II and III CC patients with either MSS or MSI‐high tumors. Although the result for stage II MSI‐high patients was not statistically significant, the small number and even lack of events in these patients suggested a potential benefit from receiving adjuvant treatment. Results from larger studies and new clinical trials will contribute to elucidate the usefulness of MSI as a predictive marker in early‐stage patients.

## Conflict of interest

The authors declare no conflict of interest.

## Author contributions

EA, JCC, HBr, and MH involved in the study concept and design. EA, LJ, HBl, MK, KT, WR, EH, JCC, HBr, and MH contributed to the acquisition of data. EA, LJ, HBl, MK, KT, WR, DB, CG, and MH analyzed and interpreted the data. EA and MH performed statistical analysis. EA and MH drafted the manuscript. HBl, EH, WR, MK, JCC, HBr, and MH obtained funding. EH, JCC, HBr, and MH provided administrative, technical, or material support. JCC, HBr, and MH underwent study supervision. All authors contributed to the critical revision of the manuscript for important intellectual content and approval of the final version of the manuscript.

## Supporting information


**Fig. S1** **.** Selection of patients for inclusion in analysis.
**Data S1** **.** Search terms for meta‐analysis.
**Fig. S2.** Balance diagnostics for overall population after adjustment for propensity score weighting.
**Table S1.** Characteristics of studies included in meta‐analysis.
