# Supplementary material for: Microsatellite instability and survival after adjuvant chemotherapy among stage II and III colon cancer patients: results from a population‐based study
Source: Mol Oncol. 2020 Jan 7;14(2):363–72. doi: 10.1002/1878-0261.12611 (PMC6998383; doi:10.1002/1878-0261.12611)
Supplement: Supplementary file 1 — Fig. S1 . Selection of patients for inclusion in analysis. Data S1 . Search terms for meta‐analysis. Fig. S2. Balance diagnostics for overall population after adjustment for propensity score weighting. Table S1. Characteristics of studies included in meta‐analysis. [file MOL2-14-363-s001.docx]

**Supplement**

**n = 2375**

patients with available information on MSI status and long-term follow-up

10 missing information on outcome

5 missing information on adjuvant chemotherapy

3 died first month

6 received neoadjuvant chemotherapy

n = 1463

878 patients with rectal cancer

34 patients with unknown location

237 stage I patients

192 stage IV patients

n = 1439

**n = 1010**

patients included in analysis

**Supplementary Figure *1****. Selection of patients for inclusion in analysis.*

**Supplementary Information:** Search terms for meta-analysis

(MSI[All Fields] OR ("microsatellite instability"[MeSH Terms] OR ("microsatellite"[All Fields] AND "instability"[All Fields]) OR "microsatellite instability"[All Fields])) AND (("colon"[MeSH Terms] OR "colon"[All Fields]) OR colorectal[All Fields]) AND ((stage[All Fields] AND ii[All Fields]) OR (stage[All Fields] AND 2'[All Fields])) AND (("mortality"[Subheading] OR "mortality"[All Fields] OR "survival"[All Fields] OR "survival"[MeSH Terms]) OR ("prognosis"[MeSH Terms] OR "prognosis"[All Fields])) AND (("drug therapy"[Subheading] OR ("drug"[All Fields] AND "therapy"[All Fields]) OR "drug therapy"[All Fields] OR "chemotherapy"[All Fields] OR "drug therapy"[MeSH Terms] OR ("drug"[All Fields] AND "therapy"[All Fields]) OR "chemotherapy"[All Fields]) OR folfox[All Fields] OR ("fluorouracil"[MeSH Terms] OR "fluorouracil"[All Fields]))


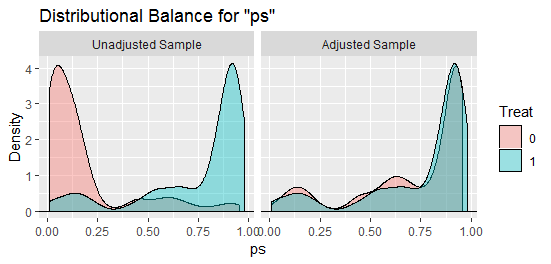


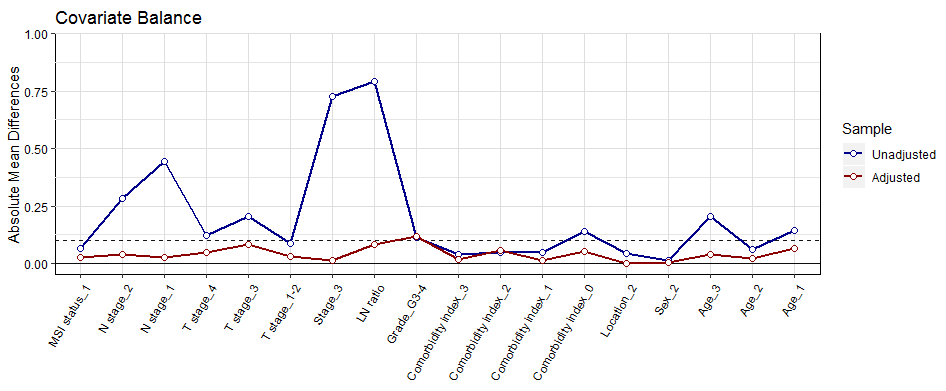


***Supplementary Figure 2.*** *Balance diagnostics for overall population after adjustment for propensity score weighting. Top: Balance plots of propensity score density for unadjusted and adjusted samples. Bottom: Absolute mean differences for covariate values among unadjusted and adjusted samples.*

**Supplementary *Table 1. Characteristics of studies included in meta-analysis***

| **Author, year** | **Study name** | **Location** | **Stage** | **N** | **n MSI (%)** | **Treatment** | **Outcomes** |
| --- | --- | --- | --- | --- | --- | --- | --- |
| Sargent, et al. 2010 | 5 randomized trials FFCD8802, NCCTG 78-48-52, NCCTG 87-46-51, INT0035, GIVIO | Colon | II and III | 457 | 70 (15%) | FU + levamisole FU + leucovorin vs surgery alone | DFS |
| Hutchins et al. 2011 | QUASAR study | Colon and rectum | II (TNM5) | 1913 | 218 (11%) | FU and folinic acid vs No chemotherapy | Recurrence rate ORs at 2 years |
| Bertagnolli et al. 2011 | CALGB 9581 and 89803 | Colon and rectum | II and III | 1852 | 330 (18%) | S2 edrecolomab or observation S3FU = leucovorin or FU/leuco/irinotec | DFS |
| Kim et al. 2015 | Cohort study Asan Medical Center | Colon | II | 860 | 126 (15%) | 5FU + leucovorin Capecitabine FOLFOX UFT, Doxyfluridine | DFS |
| Tougeron et al. 2016 | AGEO study | Colon | II and III | 433 | 433 (100%) | Surgery alone vs FU (12%) vs oxa-based (28%) | DFS |
